# Surface Modification of Stainless Steel for Enhanced Antibacterial Activity

**DOI:** 10.1021/acsomega.4c11424

**Published:** 2025-03-27

**Authors:** Metka Benčina, Niharika Rawat, Domen Paul, Janez Kovač, Aleš Iglič, Ita Junkar

**Affiliations:** † Department of Surface Engineering, Jožef Stefan Institute, Jamova 39, SI-1000 Ljubljana, Slovenia; ‡ Laboratory of Physics, Faculty of Electrical Engineering, University of Ljubljana, Tržaška 25, SI-1000 Ljubljana, Slovenia; § Department of Orthopaedics, Faculty of Medicine, University of Ljubljana, Vrazov trg 2, SI-1000 Ljubljana, Slovenia

## Abstract

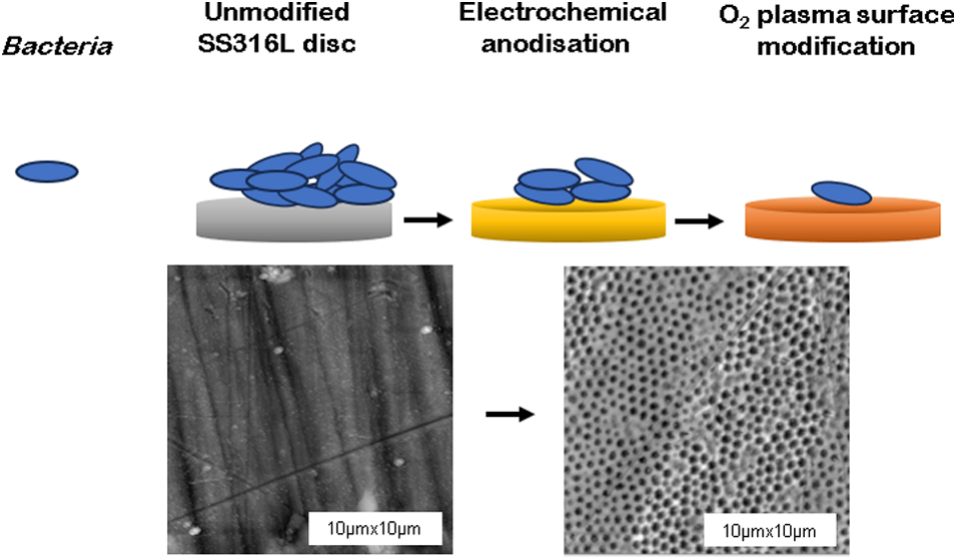

Stainless-steel grade 316L is widely used in medical and food processing applications due to its corrosion resistance and durability. However, its inherent lack of antibacterial properties poses a challenge in environments requiring high hygiene standards. This study investigates a novel surface modification approach combining electrochemical anodization and nonthermal plasma treatment to enhance the antibacterial efficacy of SS316L. The surface morphology, roughness, chemical composition, and wettability of the modified surfaces were systematically analyzed using Scanning Electron Microscopy (SEM), Atomic Force Microscopy (AFM), X-ray Photoelectron Spectroscopy (XPS), and water contact angle (WCA) measurements. SEM revealed the formation of tunable nanoporous structures with pore diameters ranging from 100 to 300 nm, depending on the applied anodizing voltage (40 and 60 V). AFM measurements demonstrated that surface roughness varied significantly with anodizing voltage, from 4.3 ± 0.4 nm at 40 V to 15.0 ± 0.6 nm at 60 V. XPS analysis confirmed the presence of Cr_2_O_3_, a key oxide for corrosion resistance, and revealed increased oxygen concentration after plasma treatment, indicating enhanced surface oxidation. Wettability studies showed that plasma treatment changed the surfaces to superhydrophilic, with WCAs below 5°. Antibacterial efficacy against *Escherichia coli* (*E. coli*) and *Staphylococcus aureus* (*S. aureus*) was significantly improved, with plasma-treated samples exhibiting up to 92% reduction in bacterial adhesion. These results demonstrate that the combined anodization and plasma treatment process effectively enhances the antibacterial and surface properties of SS316L, making it a promising strategy for applications in medical and food processing industries.

## 1 Introduction

Stainless steel is known for its resilience and relatively good corrosion resistance, making it a highly sought-after material in various industries, including biomedicine. Among the different types of stainless steel, 316L stainless steel attracts particular attention due to its biocompatibility and robust mechanical properties. Its ability to withstand harsh conditions while remaining compatible with biological systems makes it particularly suitable for medical applications, from surgical instruments to implantable medical devices. Ensuring the antibacterial activity of these medical devices is essential to preventing infections and safeguarding patient safety. Therefore, enhancing the antibacterial properties of 316L stainless steel is, therefore, of primary importance to minimize the risk of revision surgeries, reduce extensive antibiotic usage, lower medical costs, and improve overall patient outcomes.

The antibacterial activity of stainless steel surfaces can be improved through various surface modifications that either inhibit bacterial adhesion or induce bactericidal effects. It is known that surfaces with higher roughness or nanostructured features exhibit lower bacterial adhesion as the surface area increases and the surface energy changes.^1−4^ Furthermore, the topographical features of stainless steel surfaces play a crucial role in modulating bacterial behavior. For instance, nanostructured surfaces can disrupt the attachment of bacteria, may also cause so-called “contact killing” mechanisms and prevent the formation of resilient biofilms.^5−7^

Therefore, numerous methods have been explored for fabricating nanostructured stainless steel, each offering unique advantages in controlling surface morphology and, consequently, enhancing antibacterial properties. For example, physical vapor deposition (PVD) and chemical vapor deposition (CVD) techniques offer versatile approaches for depositing thin films with tailored antibacterial properties onto SS surfaces.^8^ PVD involves the deposition of atoms or molecules onto the substrate surface in a vacuum environment, while CVD involves chemical reactions to form a thin-film layer on material’s surface. These techniques allow for the precise control of film composition and thickness, enabling the incorporation of antimicrobial agents or functional coatings to enhance the antibacterial efficacy of SS surfaces. Also, laser surface modification techniques have gained attention for imparting nanostructures onto SS surfaces, improving their antibacterial performance.^9−11^ In addition, electrochemical techniques, such as anodization and electrospinning, have emerged as effective methods for precisely controlling nanofeatures (i.e., pore size) on SS surfaces.^12−14^ Nanostructuring the surface also induces changes in surface characteristics, such as increased roughness, altered wettability, and modified chemical composition, which can further influence antibacterial performance. In particular, the development of nanostructured stainless steel surfaces with antibacterial properties often involves the application of thin-film coatings^15,16^ or incorporations of certain elements, such as copper or silver, to improve their antimicrobial properties.^8,,18^

However, limited research has focused on the antibacterial effects of pure stainless steel surfaces without the addition of external elements. For instance, Erdogan and Ercan^19^ utilized anodic oxidation to create nanodimple surfaces on 316L stainless steel with feature sizes ranging from 25 to 250 nm, which resulted in increased surface area and the formation of a chromium oxide- and hydroxide-rich surface oxide layer. The nanodimpled surfaces (200 nm feature size) exhibited significant antibacterial activity, with a 71% reduction in *S. aureus* and a 58% reduction in*P. aeruginosa* colonies compared to nonanodized 316L. Similarly, a study by Rodriguez-Contreras et al.^20^ demonstrated that anodization using H_2_SO_4_/H_2_O_2_ as an electrolyte creates a nanostructured surface on SS304 stainless steel, resulting in significantly reduced bacterial adhesion, with minimal *E. coli*attachment after 1 and 4 h and a notable decrease in *B. subtilis* adhesion compared to untreated surfaces. Also, Ban et al.^5^ evaluated electropolishing and anodizing treatments on 316L stainless steel, using a mixture of phosphoric and sulfuric acids for electropolishing and an anhydrous ethylene glycol solution with perchloric acid for anodizing, and demonstrated that nanoporous surfaces with diameters of 50 and 80 nm significantly reduced bacterial adhesion (*Listeria monocytogenes*) compared to smooth surfaces.

The present study hypothesizes that nanoporous stainless steel 316L surfaces, produced via electrochemical anodization and subsequently treated with nonthermal oxygen plasma, will demonstrate improved antibacterial properties against *E. coli* (Gram-negative) and *S. aureus* (Gram-positive). This enhancement is attributed to anodization-induced alterations in surface morphology and roughness, as well as the introduction of oxygen-containing functional groups through plasma treatment, which together modulate surface chemistry and wettability.

## 2 Experimental Section

### 2.1 Materials and Methods

Ethylene glycol (Carlo Erba, for analysis), perchloric acid (Honywell, ACS reagent 70%), acetone, and absolute ethanol. A 316-grade stainless steel rod (4 mm thick, purchased from high-performance metals) was cut into specimens of discs with a diameter of 15 mm and thickness of 5 mm using a water jet cutter. Discs were hand polished and cleaned with ultrasound in ethanol.

### 2.2 Electrochemical Anodization

Electrochemical anodization was performed by Voltcraft VSP 2653 in a Teflon vessel, as a cathode Pt foil (0.1 mm thick, Premion, 99.99%, metals basis) was used. SS316L discs were used as an anode. The distance between the electrodes was 1 cm. Before anodization, specimens were cleaned by sonicating with ethanol (70%) and distilled water for 10 min each to remove surface impurities and subsequently dried in air. 5% perchloric acid was prepared in ethylene glycol and used as an electrolyte. Two distinct voltages were employed: 40 and 60 V. Meanwhile, the synthesis duration was maintained consistently at 10 min for all experiments. After the synthesis, the samples were thoroughly rinsed and ultrasonicated in absolute ethanol for 10 min. Samples were kept in absolute ethanol for 2 h, dried under the stream of N_2_ and stored inside plastic containers sealed with parafilm. The following samples were tested in further experiments: untreated SS316L (SS), plasma-treated SS316L (SS+P), SS316L anodized at 40 V (SS40), SS316L anodized at 60 V (SS60) and plasma-treated anodized samples (SS40+P and SS60+P).

### 2.3 Plasma Treatment of SS316L Discs

The treatment of SS316L discs was performed in the in-house designed plasma reactor as reported in ref^21^ Briefly; the system was evacuated with a two-stage oil rotary pump (Edwards E2M80) with a nominal pumping speed of 80 m3/h. The discharge chamber was a Pyrex discharge tube with a length of 0.8 m and an inner diameter of 0.036 m. At the center of the glass tube, a coil of six turns was mounted and coupled with a radiofrequency (RF) generator (Advanced Energy CESAR 1310) via the matching network (Dressler VM 1500 W-ICP). Gaseous inductively coupled plasma was created with a radiofrequency generator operating at a fundamental frequency of 13.56 MHz and a maximum output power of 1 kW. Commercially available oxygen with ≥ 99.9% purity was leaked into the discharge chamber with mass flow controllers (Aera FC 7700 Advanced Energy). The pressure was measured with an absolute vacuum gauge (722A MKS Baratron). Each sample was mounted into the reaction chamber in the middle of the excitation coil. After the discharge chamber was evacuated to a base pressure below 1 Pa, 5 sccm of oxygen was continuously leaked into the reaction chamber. The pressure was 75 Pa. The samples were treated for 60 s. After the plasma treatment, the samples were stored inside a tightly closed container, sealed with parafilm.

### 2.4 Characterization

#### 2.4.1 Scanning Electron Microscope (SEM) Analysis

The morphology of the surfaces was analyzed with a scanning electron microscope (JSM 7600 - JEOL) equipped with a field emission gun with an acceleration voltage of 15 kV.

#### 2.4.2 Water Contact Angle (WCA) Measurements

The wettability measurements of SS samples were performed with Drop Shape Analyzer DSA-100 (Krüss GmbH, Hannover, Germany) by a sessile drop method to measure a static contact angle. The contact angle on the surface was analyzed within 30 min after the electrochemical anodization/plasma treatment, adding a 2.5 μL drop of deionized water on 8 different surface areas. Three measurements were performed for each sample, and the average value was calculated. The relative humidity was around 45%, and the operating temperature was 21 °C, which did not vary significantly during continuous measurements.

#### 2.4.3 Atomic Force Microscopy Analysis (AFM)

Topographic features of SS samples were examined by atomic force microscopy (Solver PRO, NT MDT) in tapping mode in the air. Samples were scanned with the standard Si cantilever with a force constant of 22 N/m and at a resonance frequency of 325 kHz (the tip radius was 10 nm, and the tip length was 95 μm), and the scan rate was set to 1.3 Hz. Surface roughness was assessed through the roughness average (Ra), calculated from 10 × 10 μm^2^ images taken from 5 different measurements.

#### 2.4.4 X-ray Photoelectron Spectroscopy (XPS)

The X-ray photoelectron spectroscopy (XPS) analyses were carried out on the PHI-TFA XPS spectrometer produced by Physical Electronics Inc. Samples were put on the sample holder and introduced into the ultrahigh vacuum spectrometer. The analyzed area was 0.4 mm in diameter, and the explored depth was about 3–5 nm. Sample surfaces were excited by X-ray radiation from a monochromatic Al source at a photon energy of 1486.6 eV. XPS depth profiles were performed to obtain in-depth concentration of elements in the surface layer. Ar ion sputtering with an ion energy of 1 keV was applied. The sputtering rate was about 2 nm/min.

#### 2.4.5 Antibacterial Test

The pathogenic strains of *E. coli* and *S. aureus* were first prepared in Luria–Bertani broth for 24 h at 37 °C (Bioball, BTF, Australia). A suspensions of *E. coli*and *S. aureus* (10^5^ colonies forming unit (CFU/mL)) were prepared, from which 0.1 mL was pipetted onto the sample surfaces. The samples were then incubated in the incubator (I-105 CK UV, Kambič) for 24 h at 37 °C in a humidity box to maintain relative humidity at 90%. After incubation, bacteria (*E. coli* and *S. aureus*) on the surface were removed using 2.5 mL of sterilized phosphate-buffered saline (PBS - tablets, Sigma-Aldrich) and 0.2 mL of this solution was taken for inoculation of bacteria (*E. coli* and *S. aureus*) in the Nutrient agar plate at 37 °C for 24 h. Then the number of CFUs can be determined. For convenient counting of CFUs, before inoculating bacteria (*E. coli* and *S. aureus*) in the Nutrient agar plate, the initial solution was diluted further with PBS by a factor of 10^0^–10^5^. The CFU/mL was calculated using an automated colony counter (Acolyte 3, Symbiosis).

#### 2.4.6 Sterilization

The autoclave sterilization of samples was carried out in Autoclave (A-21CA, Kambič) for 15 min in dry mode. UV irradiation of samples was performed with UV C light (Sylvania ultraviolet G15W; 15W/cm2) for 15 min.

## 3 Results

**Figure 1:**
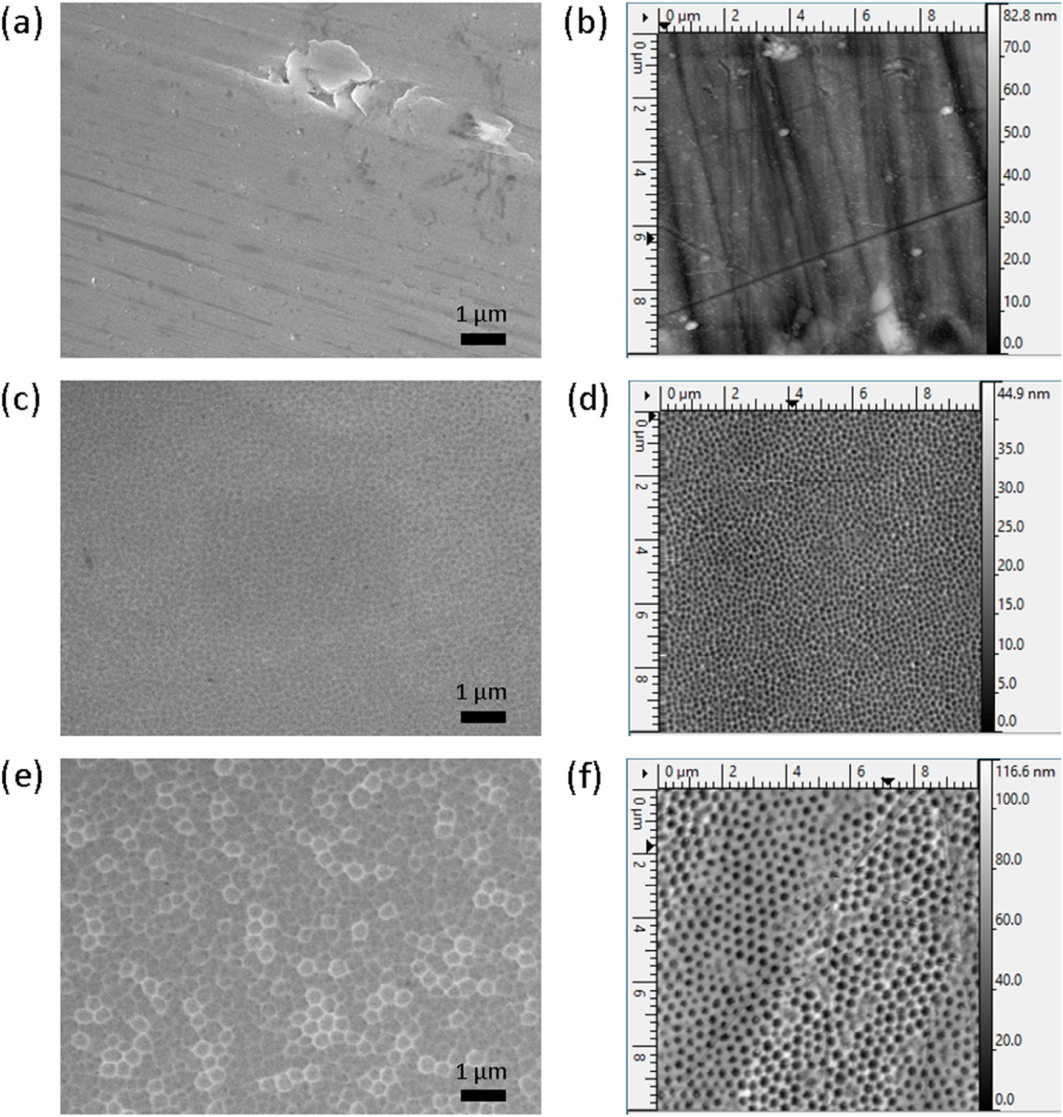
SEM and AFM images of untreated and modified SS316L, SEM image (a) and AFM image (b) of SS; SEM image (c) and AFM image (d) of SS40 and SEM image (e) and AFM image (f) of SS60.

The results of morphology analysis by Scanning electron microscopy (SEM) clearly show a correlation between the applied anodizing voltage and the development of nanopores on the surface of SS, as presented in Figure 1a,c,e. The surface morphology of the untreated SS sample (denoted as SS) shows typical features associated with the polishing process (as-purchased materials), as shown in Figure 1a. Different nanopore sizes could be obtained by altering the anodizing voltage, highlighting the tunability of the anodizing process to adjust the surface properties. In particular, the samples exposed to an anodizing voltage of 40 V (SS40) exhibited a nanoporous structure with pore diameters of 100–150 nm (Figure 1c), while the samples anodized at 60 V (SS60) had larger nanopores with a diameter of approximately 150–300 nm (Figure 1e). Subsequent SEM analysis after plasma treatment did not reveal any significant changes in the surface morphology, and therefore, these results are not included in the data set presented. Also, the Atomic Force Microscopy (AFM) results indicate a relationship between the anodizing voltage and the resulting surface roughness, demonstrating the ability to fine-tune surface characteristics through controlled anodizing processes. The AFM results provide insights into the surface roughness of variously treated SS samples, as depicted in Figure 1b,d,f. The surface roughness of the untreated SS sample (denoted as SS) is measured to be 6.3 ± 0.3 nm. For the sample anodized at 40 V (denoted as SS40), the surface roughness decreases to 4.3 ± 0.4 nm. Conversely, the sample anodized at 60 V (denoted as SS60) exhibits a significantly higher surface roughness of 15.0 ± 0.6 nm.

**Figure 2:**
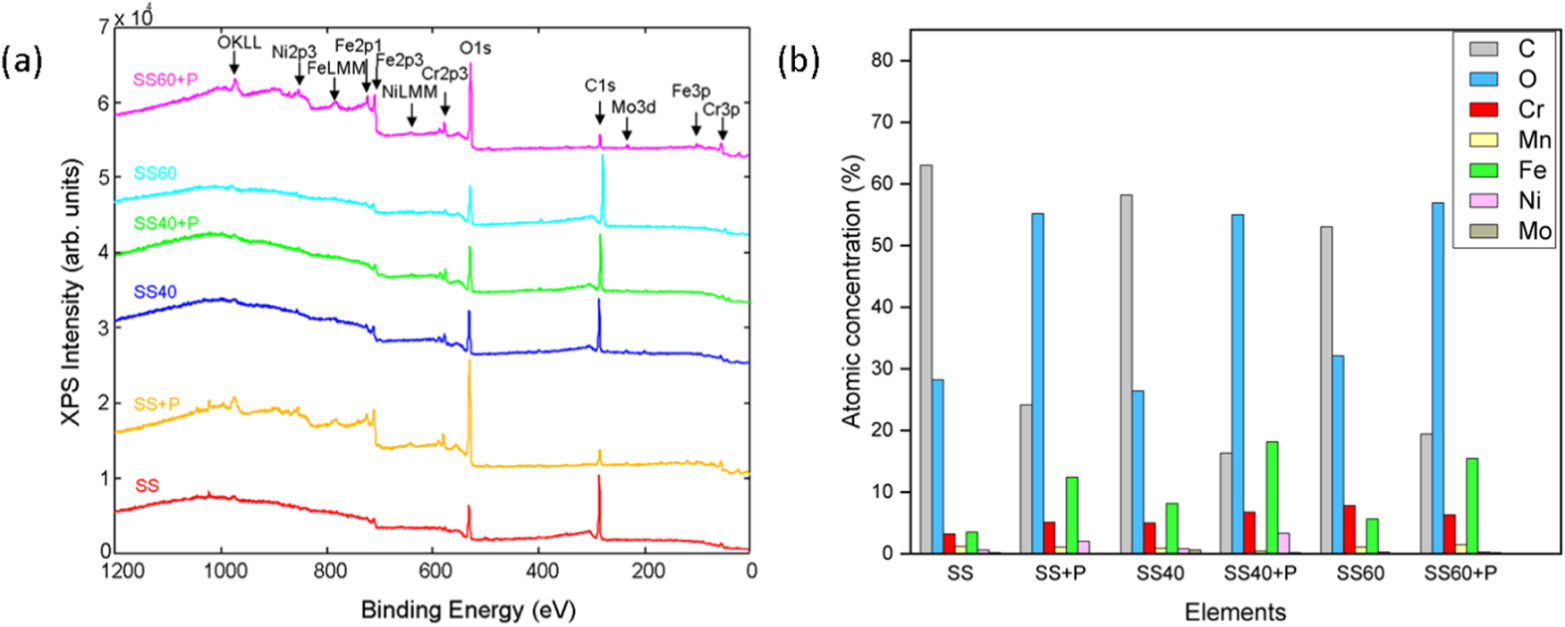
XPS survey spectra of untreated (SS), anodized (SS40, SS60), and plasma-treated samples (SS+P, SS40+P and SS60+P) (a) and elemental surface composition in at. % (b).

The chemical composition of untreated and treated SS surfaces was analyzed using X-ray photoelectron spectroscopy (XPS), as shown in Figure 2a,b. The surfaces of the SS samples are predominantly composed of elements such as carbon, oxygen, iron, chromium and nickel, with small amounts of molybdenum also detected (less than 0.9 at. %). The oxygen values remained relatively constant when comparing untreated SS with the anodized counterparts (SS40, SS60). However, the plasma-treated samples (SS+P, SS40+P and SS60+P) showed a significant increase in surface oxygen concentration (Figure 2b) due to the use of oxygen plasma treatment. At the same time, a decrease in carbon concentration was observed with an increase in iron on the plasma-treated surfaces. The anodized samples (SS40 and SS60) showed a slight increase in chromium, iron and nickel and a slight decrease in carbon compared to the untreated sample (SS). The differences in chemical composition according to XPS analysis between SS40 and SS60 were negligible, while the SS plasma-modified samples exhibited higher carbon and lower iron concentration compared to plasma anodized samples (SS40+P and SS60+P).

**Figure 3:**
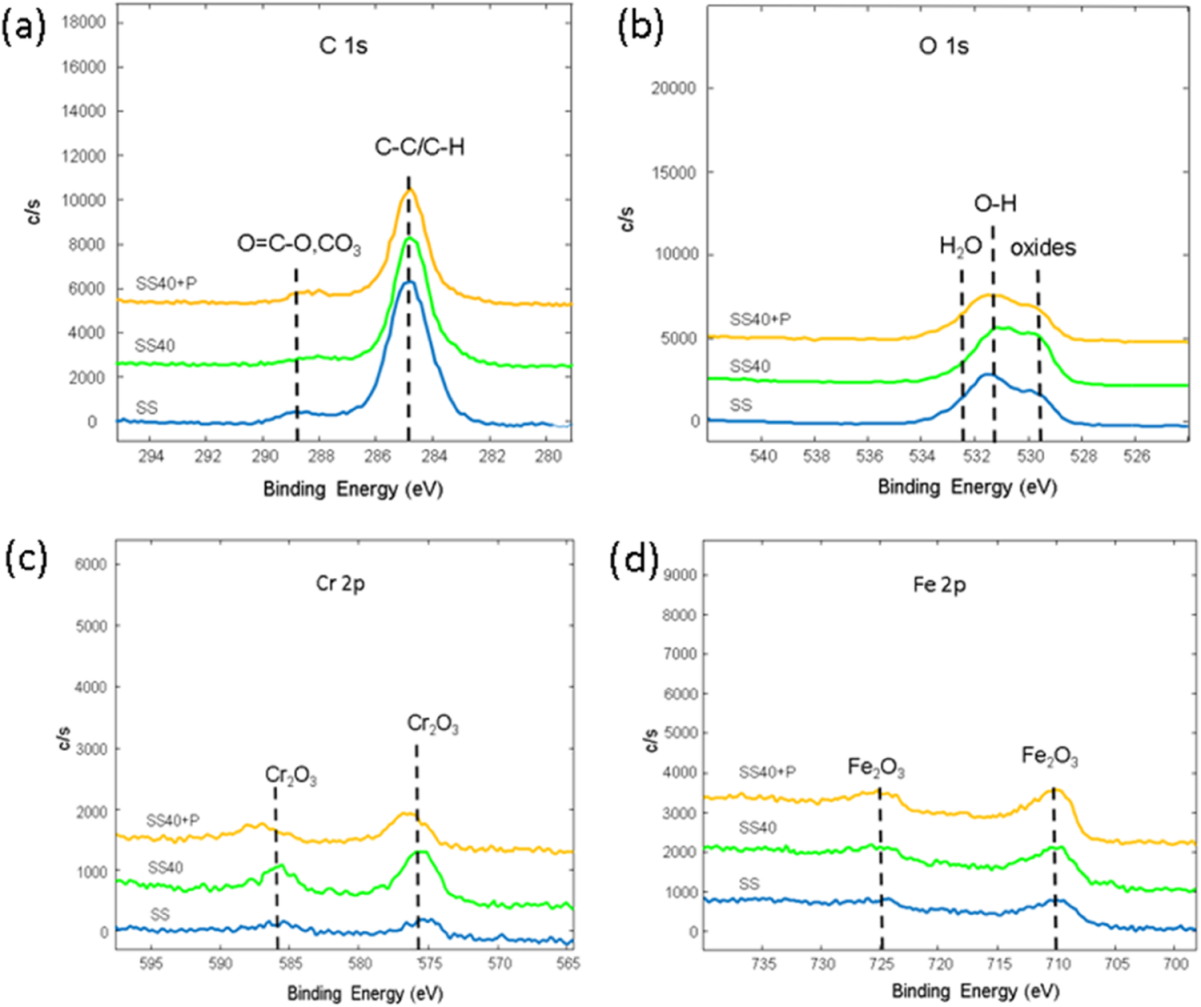
High-resolution XPS spectra of (a) O 1 s, (b) C 1s, (c) Cr 2p, and (d) Fe 2p.

High-resolution X-ray photoelectron spectroscopy (XPS) was performed to analyze the chemical bonds present in the SS samples. Examples of high-resolution C 1s spectra for untreated (SS), anodized (SS40) and anodized plasma-treated SS samples (SS40+P) are illustrated in Figure 3a. A peak dominates the C 1s spectrum at approximately 285.0 eV, indicating carbon–carbon (C–C) and carbon–hydrogen (C–H) bonds, which are likely due to surface contamination on the SS samples. A minor peak at 288.9 eV is also observed, likely associated with O=C–O and/or CO_3_ bonds from surface impurities. This peak is predominantly observed in the case of SS and SS40+P samples and can be correlated with the oxidation of the carbon layer. In the case of SS40+P, a slight decrease in the intensity of the C 1s peak is observed, suggesting the removal of organic contaminants from the surface through plasma etching.

The O 1s spectra for untreated (SS) and modified samples (SS40 and SS40+P) revealed distinct peaks, indicating the presence of various oxygen-containing functional groups (Figure 3b). A prominent peak at 529.5 eV is associated with oxygen atoms in oxide structures. In SS40+P, the peak at 529.5 eV shows an increase in intensity, which could indicate the presence of metal oxides such as Cr_2_O_3_, Fe_2_O_3_, MnO, MoO_3_, and NiO. This trend is consistent across all plasma-treated surfaces and correlates with the increased surface concentrations of Cr, Fe, and Ni (Figure 2a,b). Peaks at higher binding energies, particularly around 531 and 532 eV, are attributed to hydroxyl groups (OH−) and adsorbed water molecules, respectively.

**Figure 4:**
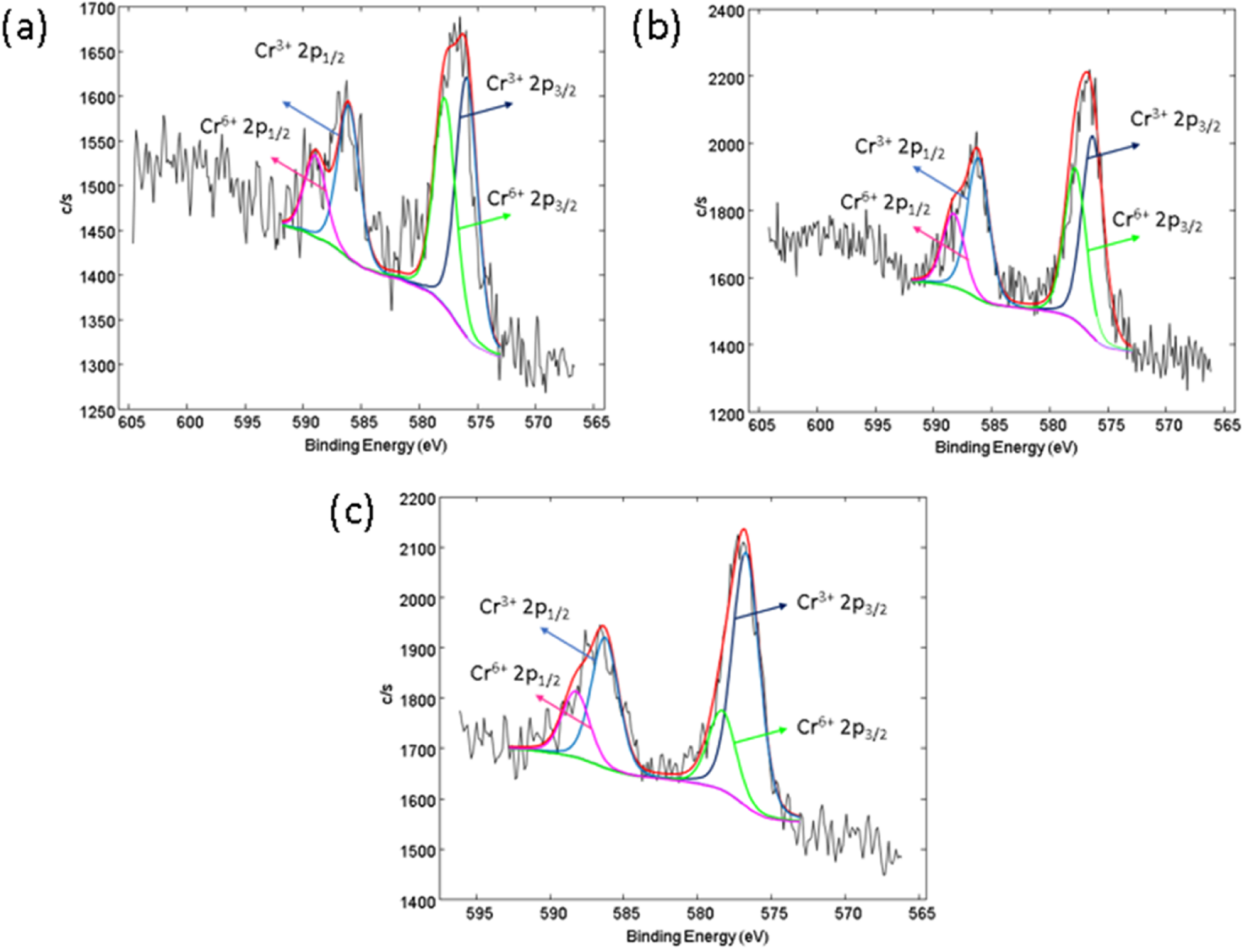
High-resolution XPS spectra of (a) Cr 2p-SS, (b) Cr 2p-SS40, and (c) Cr 2p-SS40+P.

The high-resolution XPS Cr 2p spectrum for SS samples (Figure 3c) reveals the presence of two prominent peaks detected around 577.4 6 and 586.9 eV for all samples (SS, SS40 and SS40+P), indicating the presence of chromium(III) oxide (Cr_2_O_3_)—this common and protective oxide layer forms on stainless steel surfaces. Figure 4 presents representative high-resolution photoelectron spectra for SS, SS40, and SS40+P samples after background subtraction and curve fitting using model Gaussian–Lorentzian functions. The analysis reveals the presence of Cr (VI) on the surface of SS samples, which raises significant concerns for medical applications due to its cytotoxicity and potential adverse biological effects. However, Cr (VI) is detected in lower concentrations compared to Cr (III), with a noticeable reduction in Cr (VI) content observed from SS to SS+P, indicating the effectiveness of surface modification in minimizing its presence.

Further analysis of the Fe 2p core-level spectra revealed characteristic peaks (Figure 4); the Fe 2p_3_/_2_ peak at approximately 710 eV and the Fe 2p_1_/_2_ peak at around 724 eV correspond to the Fe^3+^ oxidation state, suggesting the formation of hematite (Fe_2_O_3_).

Water contact analysis for SS samples is presented in Table 1. It can be seen that the SS exhibits an affinity for water, demonstrated by a water contact angle (WCA) of around 66°. After anodization, the water contact angle (WCA) measurements for SS40 and SS60 samples indicate hydrophobic properties. For instance, the SS40 sample shows a WCA of 63.4° ± 1.5°, and the SS60 sample exhibits an even higher WCA of 93.8° ± 3.7°. However, the introduction of plasma treatment substantially modifies the wettability of all SS samples, bringing the WCA down to under 5°, which shows a superhydrophilic nature upon the samples identified as SS+P, SS40+P, and SS60+P.

**Table 1:** Water Contact Angle (WCA) Measurements for Untreated and Modified SS316L Samples

sample	SS	SS+P	SS40	SS40+P	SS60	SS60+P
WCA (°)	66.5 ± 3	3.4 ± 2	63.4 ± 1.5	4.5 ± 2.6	93.8 ± 3.7	4.7 ± 2.9

**Figure 5:**
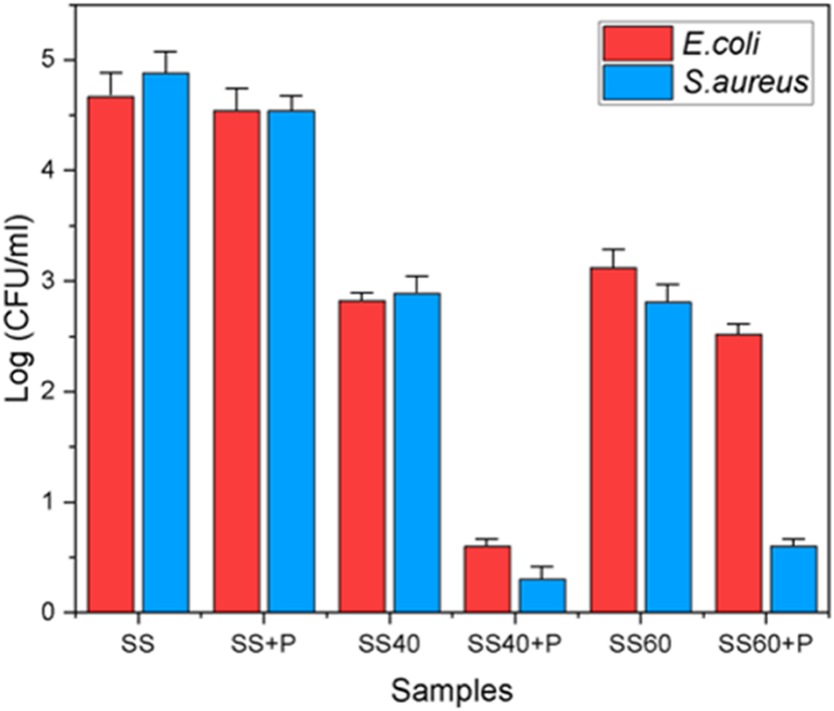
Antibacterial activity of untreated and modified SS samples.

Figure 5 illustrates the antibacterial efficacy of various surface-treated SS samples against *E. coli* and *S. aureus*, represented by the logarithmic reduction of colony-forming units (CFU) per milliliter. The untreated SS sample shows high CFU/mL for both *E. coli* and *S. aureus*, indicating limited inherent antibacterial activity. The SS+P sample exhibits a slight reduction in bacterial count for *E. coli* and *S. aureus* compared to untreated SS, respectively, demonstrating the effectiveness of plasma treatment in enhancing antibacterial properties. The SS60 V sample shows an increase in CFU/mL for both *E. coli* and *S. aureus* compared to SS40 V, which might indicate that higher voltages without plasma could potentially promote bacterial adhesion or biofilm formation. Such a result could be mainly influenced by the surface morphology of SS60 V, as the surface roughness is higher than SS40 V; the diameter of pores in this case is also increased (from about 100 to 300 nm). However, combining anodization treatment with plasma (SS40+P and SS60+P) results in reduced detectable *E. coli* and *S. aureus* on surfaces, reinforcing the effectiveness of plasma treatment in improving antibacterial performance. Notably, plasma treatment at 40 V (SS40+P) reduces *E. coli* and *S. aureus* for approximately 78 and 92%, respectively, while plasma treatment at 60 V (SS60+P) reduces *E. coli* and *S. aureus* for approximately 19 and 79%, respectively (Figure 5).

## 4 Discussion

Analysis of surface morphology reveals that anodizing of SS samples at different voltages led to the formation of distinct nanoporous structures, as shown elsewhere.^13,22^ At 40 V, nanopores with 100–150 nm diameters were observed, while anodizing at 60 V produced larger nanopores, approximately 150–300 nm. These morphological changes were accompanied by alterations in surface roughness, as revealed by AFM. The untreated SS samples exhibited a surface roughness of about 6.3 nm, while anodization at 40 V resulted in a smoother surface with a roughness of 4.3 ± 0.4 nm, linked to the formation of smaller nanopores (100–150 nm). In contrast, anodizing at 60 V significantly increased roughness to 15.0 nm, corresponding to the development of larger nanopores (150–300 nm). This demonstrates a clear relationship between pore size and surface roughness, which is consistent with findings reported in the literature. For instance, studies such as those by Herath et al.^13^ and Erdogan and Ercan^19^ have shown that anodization parameters, including applied voltage, directly influence pore size and, consequently, surface roughness; larger pores typically correlate with increased roughness. These findings align with our observations, further validating the tunability of surface properties through controlled anodization processes. The ability to control nanopore size and surface roughness through anodizing is crucial for tailoring surface properties for specific applications.^23^ Despite these substantial morphological changes, plasma treatment did not alter surface topography, as evidenced by SEM and AFM analysis (data not shown). The results suggest that plasma treatment primarily modifies the chemical composition and surface energy rather than the physical structure, which aligns with previous studies on plasma-treated surfaces.^24−26^

XPS analysis provided understanding of the chemical changes induced by anodizing and plasma treatments. Untreated SS surfaces predominantly comprise carbon, oxygen, iron, chromium, and nickel, with minor molybdenum content. Anodization increased surface concentrations of chromium, iron, and nickel, indicative of enhanced exposure of these elements due to the removal of surface contaminants and the formation of oxide layers. Plasma treatment further modified surface chemistry, significantly increasing the oxygen content while reducing surface carbon levels. This increase in oxygen is likely due to the incorporation of oxygen-rich functional groups, which also enhance surface hydrophilicity.^27^ Moreover, the reduction in carbon, particularly following plasma treatment, suggests the effective removal of organic contaminants,^28,29^ which is critical for applications that require surfaces of high cleanliness. The XPS analysis also revealed the presence of chromium(III) oxide (Cr_2_O_3_), particularly on the surfaces after anodization (example shown for SS40) and also on the plasma anodized samples (SS40+P, SS60+P), while no such increase was observed for SS+P sample. Cr_2_O_3_ is critically important on the surface of stainless steel grade 316L, especially in medical applications where corrosion resistance is important. The formation of a chromium oxide layer, primarily Cr_2_O_3_, on the surface of SS316L provides essential benefits. First, it acts as a protective barrier against corrosion,^30^ shielding the material from reacting with corrosive environments such as bodily fluids and sterilization agents. This property ensures the longevity and reliability of medical devices by reducing the risk of corrosion-induced degradation or failure. Also, Cr_2_O_3_ enhances the biocompatibility of SS316L implants by minimizing the release of metal ions into surrounding tissues, thereby mitigating potential adverse biological reactions. Cr_2_O_3_ is primarily detected on the surface of the anodized SS40 sample, and it is also notably present on the plasma-treated anodized SS40+P sample, indicating the formation of a protective oxide layer on both surfaces under different treatment conditions. Therefore, anodization and/or plasma treatment are effective in forming a Cr_2_O_3_ layer, which is essential for enhancing the corrosion resistance and surface properties of the stainless steel. In addition, XPS analysis detected the presence of Cr (VI) on the surfaces of stainless steel (SS), as shown in Figure 4. This is concerning for medical applications due to the possible toxic effects of Cr (VI). However, the results demonstrate a significant reduction in Cr (VI) concentration from untreated SS to the modified surfaces (SS40 and SS40+P), underscoring the efficacy of surface modification techniques in mitigating Cr (VI) levels. On the other hand, an increase in iron oxides (Fe_2_O_3_) is also promoted on plasma-modified surfaces, which could potentially increase the risk of corrosion. While Cr_2_O_3_ forms a protective passive layer on stainless steel surfaces and Fe_2_O_3_ is relatively stable, iron ions can still facilitate localized corrosion, particularly in chloride-rich physiological environments.^31−33^ This occurs through mechanisms such as pitting or crevice corrosion, where the breakdown of the passive layer exposes the underlying material to aggressive ions. Such corrosion processes can lead to the release of metal ions and degradation of the medical devices, potentially compromising their structural integrity and biocompatibility. Therefore, the presence of iron oxides must be carefully evaluated when employing stainless steel in medical applications.

Therefore, by optimizing surface morphology by anodization and fine-tuning surface chemistry by oxygen plasma treatment, antibacterial properties of the SS surfaces could be enhanced, which presents an interesting approach for the fabrication of antibacterial surfaces without the need to use antibacterial drugs or other antibacterial elements (like silver, copper, zinc etc.). The antibacterial tests revealed significant differences in the efficacy of treated SS surfaces against *E. coli* and *S. aureus*. Untreated SS samples showed high bacterial counts for *E. coli* and *S. aureus*, indicating limited inherent antibacterial properties. However, anodized SS surfaces (SS40 and SS60) exhibit reduced bacterial colonization, presumably due to the unique nanostructured surfaces created during the anodization process. It is known that nanostructured surfaces can kill bacteria due to cell wall rupture.^34−37^ Therefore, the anodization of SS40 and SS60 creates a surface morphology (nanopores) that could reduce bacterial viability by physically disrupting the cell walls of adhering bacteria. The plasma-treated samples (SS+P) exhibited slight reductions in bacterial counts compared to only anodized samples (SS40 and SS60), highlighting the effectiveness of plasma in enhancing antibacterial properties. This effect was more pronounced for *E. coli*, achieving almost complete eradication on SS40 V+P. For *S. aureus*, a similar trend was observed; S40 V+P significantly reduced bacterial counts, reinforcing that plasma treatment combined with anodizing enhances antibacterial performance. However, plasma treatment at 60 V (SS60+P) still reduced bacterial counts, albeit not as effectively as at lower voltages (40 V). The remarkable antibacterial efficacy observed in the plasma-treated SS samples can be attributed to chemical mechanisms disrupting bacterial adherence, which, together with specific surface morphology, enhances antibacterial efficiency. After plasma treatment, water contact angle (WCA) measurements indicate a transition of stainless steel surfaces from hydrophobic to superhydrophilic; the near-zero WCA observed in plasma-treated samples (SS+P, SS40+P, SS60+P). There is a lack of literature on the antibacterial activity of nonthermal plasma treatment of nanostructured stainless steel 316L. However, nonthermal plasma alters the surface chemistry by introducing oxygen-containing functional groups, which can affect the antibacterial performance of stainless steel surfaces. These functional groups, such as hydroxyl (−OH) and carbonyl (C=O), increase the surface energy and hydrophilicity of the SS. A more hydrophilic surface is less conducive to bacterial attachment because bacteria tend to adhere more easily to hydrophobic surfaces where they can form biofilms.^38^ Similar findings were reported by Bruzaud et al.,^39^ which showed that *L. monocytogenes* and *P. aeruginosa* are repelled by superhydrophobic stainless steel surfaces.^39^ Also, DeFlorio et al.^40^ demonstrated that electroplated nickel-nanodiamond coatings functionalized with organosilane molecules impart superhydrophobicity to 304 stainless-steel surfaces, significantly reducing bacterial adhesion of *E. coli*and *L. innocua*. Besides altering the surface wettability, plasma treatment generates reactive oxygen species (ROS), such as atomic oxygen, ozone, and radicals, with strong antimicrobial properties.^41^ These ROS can damage bacterial cell components, including lipids, proteins, and DNA, leading to oxidative stress and cell death.^42−45^

To sum up, the combination of anodizing and plasma treatment produces a synergistic effect that amplifies the antibacterial properties of SS. While anodizing creates a nanostructured surface that physically disrupts bacterial cells, plasma treatment modifies the chemical composition and wettability to create an environment that is hostile to bacterial survival. However, in biomedical applications, the long-term behavior of anodized and plasma-treated stainless steel in a physiological environment, particularly regarding protein adsorption and antimicrobial protection, needs further consideration. As observed by Zhao et al.,^46^ proteins can adsorb onto nanostructured surfaces, alter their geometry and, in some cases, mask functional groups critical for bactericidal performance. Future studies examining the impact of protein interaction and long-term exposure to physiological conditions are essential to fully assess the suitability of anodized and plasma-treated SS for extended use in biomedical applications.

## 5 Conclusions

This study presents a novel approach combining electrochemical anodization and oxygen nonthermal plasma treatment to enhance the antibacterial properties of stainless steel 316L (SS). Nanostructured surfaces with nanopore diameters of 100–150 nm (SS40) and 150–300 nm (SS60) have been fabricated by systematically tuning the anodization voltage. Surface chemistry modification through plasma treatment further introduced oxygen-rich functional groups, significantly increasing surface hydrophilicity, as evidenced by a reduction in water contact angle from 66.5° ± 3.0° (untreated SS) to below 5° (plasma-treated SS40+P and SS60+P). Antibacterial tests demonstrated that SS40+P exhibited the highest efficacy, reducing *E. coli* and *S. aureus* by approximately 78 and 92%, respectively, compared to untreated SS. SS60+P also showed notable antibacterial activity, reducing *E. coli*and *S. aureus* by 19 and 79%, respectively. The improved antibacterial performance can be attributed to the synergy between nanostructured surface morphology, which physically disrupts bacterial cells, and plasma-induced chemical modifications, which increase hydrophilicity and introduce reactive oxygen species that inhibit bacterial adhesion and proliferation.
